# Considerations regarding a diagnosis of Alzheimer’s disease before dementia: a systematic review

**DOI:** 10.1186/s13195-022-00971-3

**Published:** 2022-02-10

**Authors:** Jetske van der Schaar, Leonie N. C. Visser, Femke H. Bouwman, Johannes C. F. Ket, Philip Scheltens, Annelien L. Bredenoord, Wiesje M. van der Flier

**Affiliations:** 1 Alzheimer Center Amsterdam, Department of Neurology, Amsterdam Neuroscience, Vrije Universiteit Amsterdam, Amsterdam UMC, De Boelelaan 1118, 1081 HZ Amsterdam, The Netherlands; 2 Division of Clinical Geriatrics, Center for Alzheimer Research, Department of Neurobiology, Care Sciences and Society, Karolinska Institutet, Stockholm, Sweden; 3 Medical Library, Vrije Universiteit, Amsterdam, The Netherlands; 4 Erasmus School of Philosophy, Erasmus University Rotterdam, Rotterdam, The Netherlands; 5 Department of Epidemiology & Data Sciences, Vrije Universiteit Amsterdam, Amsterdam UMC, Amsterdam, The Netherlands

**Keywords:** Alzheimer’s disease, Biomarkers, Diagnosis, Disclosure, Preclinical, Prodromal, Predementia, Ethics

## Abstract

**Background:**

The NIA-AA research framework proposes a purely biological definition of Alzheimer’s disease (AD). This implies that AD can be diagnosed based on biomarker abnormalities, irrespective of clinical manifestation. While this brings opportunities, it also raises challenges. We aimed to provide an overview of considerations regarding the disclosure of AD pathology before the onset of dementia.

**Methods:**

A systematic literature review was conducted and reported according to PRISMA guidelines. We searched PubMed, Embase, APA PsycINFO, and Web of Science Core Collection (on 10 December 2020) for references on conveying AD biomarker results to individuals without dementia. Our query combined variations on the terms Alzheimer’s disease, disclosure, or diagnosis, preclinical or prodromal, and biomarkers. Two reviewers independently screened the resulting 6860 titles and abstracts for eligibility and examined 162 full-text records for relevance. We included theoretical articles in English, on communicating amyloid and/or tau results to individuals with mild cognitive impairment, subjective cognitive decline, or normal cognition. MAXQDA-software was used for inductive data analysis.

**Results:**

We included 27 publications. From these, we extracted 26 unique considerations, which we grouped according to their primary relevance to a clinical, personal, or societal context. Clinical considerations included (lack of) validity, utility, and disclosure protocols. Personal considerations covered psychological and behavioral implications, as well as the right to (not) know. Finally, societal considerations comprised the risk of misconception, stigmatization, and discrimination. Overall, views were heterogeneous and often contradictory, with emphasis on harmful effects.

**Conclusions:**

We found 26 diverse and opposing considerations, related to a clinical, personal, or societal context, which are relevant to diagnosing AD before dementia. The theoretical literature tended to focus on adverse impact and rely on common morality, while the motivation for and implications of biomarker testing are deeply personal. Our findings provide a starting point for clinicians to discuss biomarker-based diagnosis with their patients, which will become even more relevant in light of the conditional approval of a first disease-modifying drug for AD.

## Introduction

The pathophysiological cascade of events in Alzheimer’s disease (AD) starts 20 to 30 years before dementia [1–3]. Nowadays, it is possible to detect this pathology in vivo, using biomarkers. The National Institute on Aging and Alzheimer’s Association research framework operationalized AD as a biological construct characterized by evidence of amyloid plaques, tau tangles, and neurodegeneration, irrespective of clinical expression [4]. This implies that AD can be diagnosed before dementia, in individuals with mild cognitive impairment, subjective cognitive decline, or normal cognition.

This development has sparked a heated debate. Should a specialist in a memory clinic tell individuals without dementia that they have AD? This may lead to distress [5] as a precise prognosis cannot be given and there is no curative therapy (yet). Alternatively, can physicians withhold information on an underlying disease from patients, just because they do not fulfill clinical dementia criteria? While perhaps avoiding anxiety, this would deprive patients of the opportunity to adopt a risk-reducing lifestyle, prepare for the future, or participate in dementia-prevention trials [6].

The numbers of persons living with preclinical AD are large [7] and many wish to learn their biomarker results [8]. A first estimation suggests the prevalence of AD may be three times higher when based on a biological rather than a clinical definition of the disease, illustrating the potential magnitude of consequences [9]. With the conditional approval of a first disease-modifying therapy [10], this discussion is more relevant than ever.

We aim to provide an overview of ethical, psychosocial, and societal considerations regarding the disclosure of AD pathology before dementia.

## Methods

A systematic literature search was conducted and reported according to PRISMA guidelines [11]. Our broad query combined synonyms and spelling variations on the terms “Alzheimer’s” AND “disclos*” OR “diagnos*” AND “predementia” AND “biomarkers,” using controlled standardized keywords as well as free text terms. We searched PubMed, Embase, APA PsycINFO, and Web of Science Core Collection for references published before 10 December 2020 and scanned reference lists of identified articles.

All articles in English presenting theoretical data, i.e., ethical concerns, psychosocial consequences, and societal implications of disclosing amyloid and/or tau results to individuals in AT(N) stages 1–3 [4] were eligible (provided the full text was available), except (sections of) books, editorials, commentaries, and conference proceedings. Publications on later stages and other types of dementia or neurodegenerative diseases were excluded, as well as those primarily focused on trial design or genetic risk.

Two authors independently screened all titles and abstracts. Articles marked as potentially relevant were assessed for eligibility based on full text. In case of discrepancy, arguments for inclusion and exclusion were discussed while re-examining the contents and criteria. In each case, consensus was reached, without having to consult a third author.

We performed inductive content analysis [12], using MAXQDA-software. First, all sections addressing ethical, psychosocial, and societal considerations regarding the disclosure of AD pathology before dementia were identified. Next, the data were processed in an iterative and incremental manner to highlight all aspects pertinent. These were further grouped and aggregated in a hierarchy of categories. This process was repeated and revised until the contents were precisely described and fully covered, by a structure of considerations, with underlying arguments and overarching contexts. An additional classification was made according to the key ethical principles of medical ethics: beneficence, non-maleficence, justice, and autonomy [13].

## Results

**Fig. 1:**
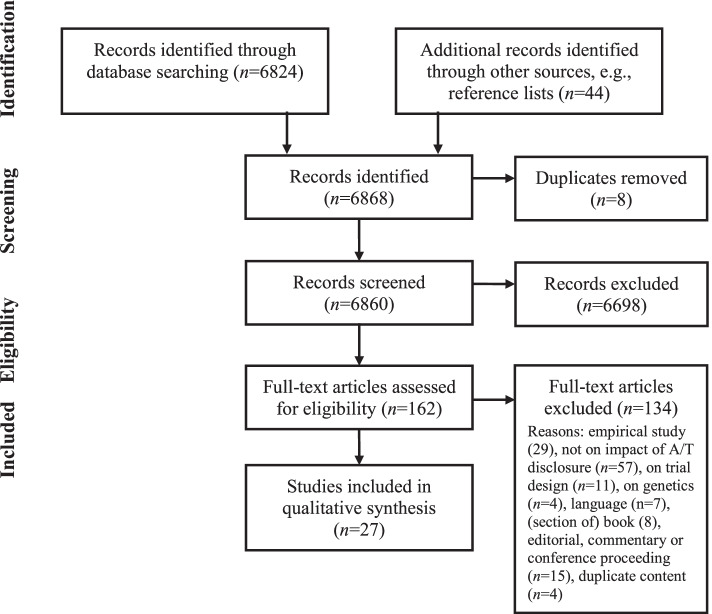
Flow diagram of study selection

Our initial search yielded 6860 records. Two reviewers independently screened all titles and abstracts for eligibility and examined 162 full-text records for relevance. After applying selection criteria (Fig. 1), we included 27 articles.

From these, 26 unique considerations were extracted (Table 1), along with supporting arguments, and further categorized according to their primary relevance to a clinical, personal, or societal context. The final coding tree corresponds to the structure of Table 1.

**Table 1:** Clinical, personal, and societal considerations

	Consideration	Refs	Arguments
	**Clinical**		
1	Validity	[14–28]	• Clinical criteria have limited validity • Biomarkers enhance diagnostic certainty and accuracy • Validity is strong or sufficient (in selected cohorts) • Predictive value for progression is demonstrated (in selected cohorts)
2	Lack of validity	[14–39]	• Validity is uncertain and insufficient or needs further research • Predictive value is uncertain and limited or needs further research • Discerning normal aging from latent disease is difficult or impossible • Many individuals with AD biomarkers never develop dementia • Abnormal biomarkers are not the sole cause of AD • Procedures may be burdensome or risky • Consequences of misdiagnosis are severe
3	Utility	[14, 16–19, 21–24, 26–32, 34–36, 40]	• Drugs can suppress symptoms in some patients • Lifestyle changes or interventions may be beneficial • Early diagnosis may lead to better healthcare
4	Lack of utility	[14–18, 20–24, 26–32, 34–36, 38, 40]	• Utility is absent and uncertain or needs further research • There is no disease-modifying therapy • Lifestyle changes or interventions have uncertain or modest effect at best • Lifestyle changes should be done regardless
5	Protocols and training	[14, 16–18, 20–23, 25, 26, 28, 30–32, 36–40]	• Protocols, methods, and materials are scarce or required • Knowledge of genetic or oncological markers may offer a starting point • Healthcare professionals need to develop knowledge and skills
6	Disclosure	[14–25, 27–32, 34–40]	• There is growing consensus toward the use of biomarkers and sharing of results • Disclosure is difficult, especially to a person with (full) insight • Safety may be improved by informed consent, pre-test counseling, post-disclosure support, take-home materials, time for reflection, involvement of a relative, and tailoring the approach to the individual’s needs • Research into the impact of disclosure is scarce or required
	**Personal**		
7	Certainty	[14, 16, 17, 20, 23, 24, 28, 34, 37, 39]	• An in vivo (biomarker) diagnosis of AD is inherently uncertain • Individuals may appreciate an uncertain risk prediction • Individuals may understand uncertainty
8	Uncertainty	[14, 15, 17, 20, 22–25, 28, 31, 32, 34–36]	• Early detection may lead to more or longer uncertainty • Individuals may expect a certain diagnosis • Individuals may misinterpret test results • It is hard to deal and live with uncertainty
9	Actionability (personal utility)	[14–25, 27, 28, 31, 32, 34–39]	• Individuals and relatives can prepare for the future • Individuals can advance plans or improve quality of life • Individuals can contribute to or profit from prevention trials
10	Lack of actionability (personal utility)	[15, 16, 23, 25, 28, 32, 34]	• There is no actionability • Results may be too uncertain for decisions • Preparing for the future should be done regardless
11	Positive psychological impact	[14–20, 22–24, 27, 32, 36]	• Result may lead to positive emotions, e.g., relief, solace, or social exoneration (by providing an explanation for behavior or functioning) • There is value in knowing, understanding, and accepting the situation
12	Negative psychological impact	[14, 16–18, 20–32, 34–40]	• Result may lead to negative emotions, e.g., fear, anxiety, and depression • There is risk of catastrophic reactions (euthanasia or suicide) • Negative reactions may be over-rated, limited, or preventable • Early detection may increase (subjective) cognitive decline (stereotype threat)
13	Right to (not) know	[14, 15, 17, 19–25, 27–35, 39]	• Individuals have a right to know their status in research and the clinic • Individuals have a right to not know their status in research and the clinic • Right to (not) know may be overruled by the principle of *primum non nocere* (first, do no harm)
14	Wish to (not) know	[14–22, 24, 25, 28, 29, 31, 32, 35, 36, 38, 39]	• Many individuals wish to know • Some individuals do not wish to know • Individual’s preference should be included in the informed consent
	**Societal**		
15	Share	[14, 16, 19–21, 23, 24, 28, 30–33, 35, 37–39]	• Patients have the right to privacy and confidentiality • Fear of stigma and discrimination may prevent individuals from seeking help • A predementia diagnosis may lead to support
16	Support	[14, 20, 22, 28, 30, 32, 37]	• Services are needed to help individuals cope with predementia AD
17	Stigma	[14, 16, 20–26, 28–30, 32, 34, 35, 37, 38, 40]	• Label of AD may lead to loss of status, identity, or personhood • Label of AD may lead to public stigma, e.g., pity, patronizing, and distancing • Label of AD may lead to self-stigma • Label of AD may lead to spillover stigma (extends to relatives) • Predementia detection may increase the stigma of AD • Predementia detection may decrease the stigma of AD
18	Discrimination	[14, 16, 17, 20–24, 27–33, 35, 37–40]	• Predementia detection may lead to discrimination in employment, insurance, rights (voting and driving), health care, legal status
19	Policy and law	[14, 20–25, 28, 30–33, 35, 37–39]	• Current laws do not protect individuals from biomarker discrimination • Regulation and law should regulate confidentiality and privacy • Regulation and law should regulate obligation to (not) disclose • Regulation and law should regulate forced screening or testing
20	Misconception	[14–17, 19–25, 27–32, 34–38]	• The concept of predementia AD is difficult to explain and understand • (Re) conceptualization of AD may lead to misconceptions • At-risk individuals are prone to (therapeutic) misconceptions • Healthcare professionals are prone to misinterpretation • The general public is prone to misconceptions
21	Engagement and education	[14, 16, 20–22, 24–26, 28, 30–32, 35, 37–39]	• Patients and the public should be involved in the design of protocols and policy • Education may improve awareness, acceptance, and attitudes
22	Advance research	[14–16, 18, 20, 22, 23, 27–35, 37, 38, 40]	• Therapies are more likely to prevent than cure AD • Predementia phase offer opportunities to stop, delay, or slow symptom onset • Potential interventions will target patients in early stages of the disease
23	Medicalization	[14, 20, 23, 28, 32, 34, 35, 37]	• Predementia detection may lead to medicalization and overdiagnosis • Predementia detection may increase urgency or treating AD • Predementia detection may decrease urgency or treating AD
24	Resources	[14, 16–18, 20, 22–24, 27, 28, 30–33, 35, 37, 39]	• Tests and treatment may not be affordable or accessible for all • Predementia detection may exhaust healthcare and overwhelm current systems • Predementia detection may prioritize prevention over care or other research • Early detection may lead to higher cost • Early detection may lead to lower cost
25	Hope	[23, 28, 34]	• Research (participation) should not be motivated by false hope
26	Fear	[14, 16, 23, 24, 26, 29, 31, 32, 34, 35, 37, 39]	• There is a lot of fear for (the implications) of AD • Predementia detection may increase fear

Considerations according to context. By inductively analyzing the literature presenting theoretical data on disclosing the presence of AD pathology to individuals without dementia, we extracted 26 unique considerations from 25 articles. We categorized these considerations according to the context they primarily related to, i.e., clinical, personal, and societal context, and collected the underlying arguments as stated by the authors

### Clinical considerations

#### (Lack of) clinical validity

Although most authors acknowledged that biomarker information enhances diagnostic accuracy [14–25] and validity is strong in selected cohorts [17–21, 26, 27], the predictive value was debated [14–36]. About half of the articles covered the difficulty to discern normal aging from latent AD [14–16, 18, 20, 22, 26, 27, 29–31, 33, 34, 37], as the majority of cognitively healthy older persons with abnormal biomarkers never develop dementia [14–16, 20–23, 26, 27, 29–31, 33, 34, 38], since AD is multi-factorial [16, 18, 26, 27, 31, 35]. It was argued that procedures are not without burden or risk [14, 16, 18, 20, 21, 23, 24, 34] and consequences of incorrectly labeling people as “patients in waiting” [34] could be severe for the individuals and relatives concerned [14, 16, 24–28, 31, 36]. Most authors concluded that biomarker criteria require final demonstration of validity in populations without dementia [14–23, 26, 27, 30, 31, 33–36, 39].

#### (Lack of) clinical utility

Clinical utility was contested, mainly due to the absence of a disease-modifying therapy [14–16, 18, 20, 21, 23–25, 27, 28, 31, 34–36, 38, 40], and limited effectiveness of symptom suppressing medications, which has not been demonstrated in predementia stages of AD [21, 24, 40]. However, several authors suggested lifestyle interventions could delay cognitive impairment [14, 16, 17, 21–32, 34, 36], although evidence on their effectiveness remains inconclusive [14, 15, 21, 31, 36], and according to some, such health improvements should be pursued regardless of one’s biomarker status [16, 23, 31]. Lastly, it was argued that early detection can improve patient care, e.g., by offering an explanation for concerns, anticipating medical needs, and facilitating access to support [14, 16, 18, 19, 22–25, 27, 28, 32, 35]. Nonetheless, others did not consider biomarker information medically meaningful [15, 16, 20–26, 29–32, 36, 40].

#### Protocols and training

A dozen papers addressed the need for guidance regarding who to test, what findings signify, whether to disclose, and how [17, 18, 20–22, 25, 28, 31, 36–39]. Knowledge from the fields of oncology and genetic testing may offer a good starting point [14, 20, 23, 25, 27, 28, 30, 36–40]. The literature referred to the development of standardized processes and materials for disclosing biomarker levels in prevention trials [17, 30, 32, 37, 38], but reported “a complete lack of studies in a clinical setting” [17]. In addition, it was emphasized that the required skills cannot be assumed, so medical professionals would profit from training in risk communication [14, 16, 18, 20, 22, 25, 28, 37, 38].

#### Disclosure

Conveying an AD diagnosis was considered daunting, especially to persons with full insight [15, 17, 20–24, 29, 30, 40], as knowledge of the impact is scarce, yet urgently required [14–17, 20, 22–25, 27, 30–32, 34, 37, 38]. Recommendations derived from research on genetic risk covered informed consent, pre-test screening, education and counseling, sufficient time for consultation and reflection, take-home materials, involving a relative, and tailoring to individuals [14, 15, 17, 19–22, 24, 25, 27, 28, 30–32, 35–39].

### Personal considerations

#### (Un)certainty

While an abnormal biomarker result provides certainty on the presence of brain lesions, it was also argued to cause uncertainty about the eventuality of cognitive decline [14, 20, 23–25, 32, 34], which was suggested to be hard to deal with [20, 21, 32]. While a few authors felt individuals might appreciate and understand such indefinite risk [17, 23, 34, 37, 39], the majority feared that it would be misinterpreted as an inevitability [14, 15, 20, 22, 24, 28, 31, 35, 36].

#### (In)actionability

Proponents stated that awareness of having AD without dementia allowed individuals and relatives to prepare for the future by arranging private, professional, financial, and legal matters; obtaining long-term care insurance; writing advance directives or making end-of-life decisions [14–25, 27, 28, 31, 32, 34, 36–39]; participating in prevention trials [14–18, 20, 22–24, 35, 38]; or retiring early and enjoying time left [14, 23]. Opponents believed these things should be done anyway [15, 16, 23] and questioned whether the predictive power was sufficient to substantiate far-reaching decisions [15, 25, 28, 32].

#### Positive and negative psychological impact

Several publications observed the absence and even presence of AD lesions might offer relief, solace, or an explanation of symptoms [17–19, 22–24, 27, 32, 36]. Knowing what is going on was thought to be of value in itself [14–17, 20, 22–24, 27, 32]. Yet, nearly all papers mentioned adverse emotions, including fear, anxiety, and depression [14, 16–18, 20–32, 34–39], or catastrophic reactions, e.g., suicide [17, 18, 20, 22, 23, 25, 28, 30, 32, 35, 36, 38–40], despite reason to believe negative reactions may be over-rated, limited, or temporary [17, 18, 20, 22, 23, 25, 28, 30, 32, 35, 36, 38, 39]. Another worry was the risk of stereotype threat or nocebo reaction, where the knowledge of susceptibility leads to the associated behavior or a decrease in memory functioning [14, 17, 23, 28, 31, 34, 37].

#### Right to (not) know

On moral and legal grounds, an individual’s request to access one’s personal data must be granted, be it in a research trial or clinical practice [15, 17, 19–25, 27–29, 31, 34, 39]. Likewise, a refusal to be informed of such information must be respected as well [14, 15, 20, 21, 23–25, 28, 35]. However, it was argued that this fundamental right can be in conflict with physicians’ oath of *primum non nocere* (first, do no harm) and in some cases could or even should be overruled [14, 17, 19–24, 27–33].

#### Wish to (not) know

Authors reported that many individuals express a desire to receive risk information [15–17, 22, 24, 25, 28, 29, 31, 32, 36, 39], while some might prefer to remain ignorant of uncertain odds [16, 17, 21, 24, 29, 32]. It was recommended that extensive and “truly” informed consent [31] should record a patient’s preference and list which other persons and authorities will be notified [14, 15, 18, 20, 21, 24, 28, 31, 32, 35, 38, 39].

### Societal considerations

#### Sharing

As individuals have the right to privacy and confidentiality [14, 19–21, 23, 24, 28, 30–33, 35, 37–39], it was emphasized that diagnostic information should not be released to relatives [24, 35] or third parties [14, 20, 21, 23, 24, 30–33, 35, 37, 39] without their consent and against their interest, although in case of driving, physicians may be obliged to report this to relevant authorities [38]. It was also mentioned that fear of stigma could prevent patients from voicing their concerns and seeking help, while acting on their worries and needs may also yield support [14, 16, 20, 23, 28, 32, 35, 37].

#### Support services

Apart from pre- and post-diagnostic counseling [14, 17, 20–22, 24, 28, 30–32, 35, 37–39], literature addressed the need of support services for people along the continuum of AD, including assistance in personal, social, and healthcare needs and monitoring of professional, financial, and legal capacities [14, 20, 22, 26, 28, 30, 32, 37, 39].

#### Stigma

According to the identified literature, public stigma ranges from patronizing attitudes to social distancing, exclusion, and isolation [14, 23, 25, 26, 28–30, 32, 34, 37, 38, 40]. This induces self-stigma, when pejorative views are internalized as feelings of shame, lowered self-esteem, and inferiority [14, 23, 30, 32, 34, 37]. In addition, spillover stigma is detrimental to family members [14, 28, 29, 32, 37]. An increase of predementia patients was expected to expand stigma [14, 23, 30, 34, 37, 38]; conversely, normalization was assumed to dilute it as well [14, 20, 37].

#### Discrimination

Individuals with an AD diagnosis and risk of dementia were considered vulnerable to discrimination, affecting their professional position, insurance fees, legal status, civil rights (driving and voting), and financial capacity [14, 16, 17, 20–24, 27–33, 35, 37–40].

#### Policy and law

Current legislation, such as the United States Genetic Information Non-Discrimination Act and the Americans with Disabilities Act, does not adequately protect individuals with predementia AD [20, 22, 28, 33, 37, 38]. Several authors advocated for regulation of confidentiality and privacy, preclinical screening, and obligatory disclosure for persons with high responsibility [14, 20, 21, 23–25, 28, 30–33, 35, 37–39].

#### Misconception

Authors observed that a symptomless condition with an uncertain prognosis is hard to grasp for lay persons and medical professionals alike [14, 17, 20, 22–24, 28, 29, 35, 36], and the changing meaning of “AD” could lead to incorrect interpretations [14, 21, 24, 29, 30, 32, 34], especially since at-risk individuals are prone to misconceptions [14–17, 22, 23, 28, 31, 34–36], healthcare providers apply different interpretations of disease criteria [14, 16, 17, 19, 22, 27–29, 31], and the general public is influenced by dementia myths and the media’s portrayal of AD patients as “dehumanized shells” [14, 25, 30, 37].

#### Education and engagement

Several publications stress that individuals’ perceptions can change after intervention or experience [15, 17, 28, 31, 34, 38], and public dialogue may improve awareness and attitudes [14, 22, 24–26, 28, 30, 31, 35, 37, 39]. Moreover, patients of all cultures should be involved in the development of protocols and policy, to represent their own views, improve research, and decrease stigma [14, 16, 20–22, 24, 25, 28, 30, 32, 35, 37, 40].

#### Resources, opportunities, and costs

Authors worried that predementia testing may not be accessible and affordable for all [14, 16, 23, 24, 27, 30, 37], individuals with minimal symptoms could strain healthcare services [14, 22, 23, 37], and a focus on prevention research might come at the expense of patients with advanced AD [14, 16, 20, 35, 37]. Thus, the emotional and financial burden could rise substantially [14, 16–18, 20, 23, 24, 30–33, 35, 37] or drop considerably when patients live longer at home, at-risk participants lower trial costs, and medication becomes available [14, 16, 18, 20, 23, 27, 28, 32, 39].

#### Medicalization

Expanding the criteria for AD raised concerns of tipping the scales from under- to overdiagnosis [14, 20, 23, 28, 32, 35, 37]. Paradoxically, normalization was reasoned to result in marginalization, but also argued to increase the urgency to develop disease-modifying therapies [14].

#### Advance research

The primary consideration behind the new criteria is to prevent individuals with AD from developing dementia, as early interventions are hypothesized to have better chances of success [14–16, 18, 20, 22, 23, 27–35, 37, 38, 40].

#### Hope and fear

Finally, authors were wary of inflating unsubstantiated hope [23, 28, 34] and/or further fueling already widespread fear [14, 16, 23, 24, 26, 29, 31, 32, 34, 35, 37, 39]. Both were primarily regarded as vulnerabilities and impediments to rational decision-making [23, 34, 35].

### Key principles

**Fig. 2:**
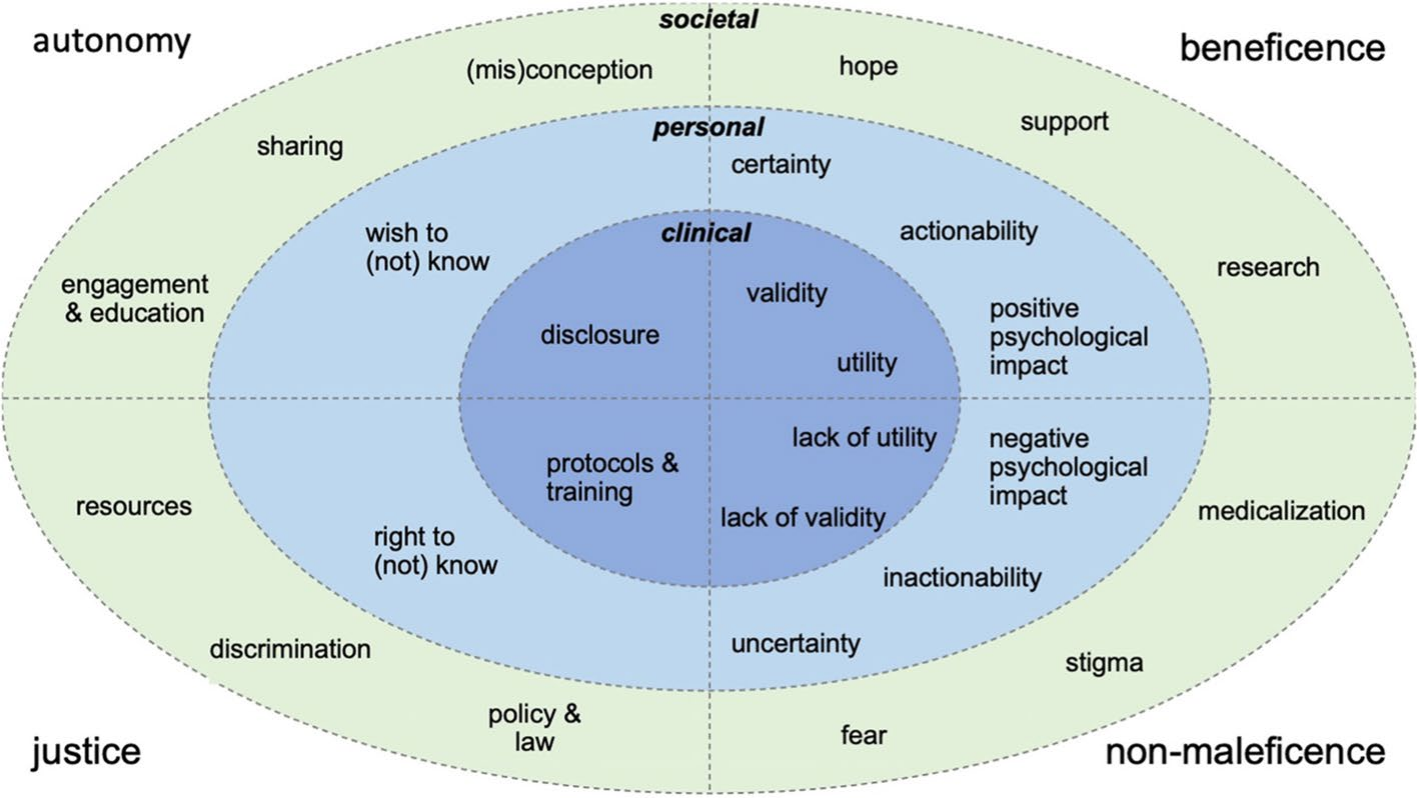
Visual overview of considerations. Visual overview of 26 considerations extracted from the included literature, categorized based on the clinical, personal, or societal context they relate to and the four basic principles of biomedical ethics: beneficence (doing good), non-maleficence (avoiding harm), justice (ensuring fair distribution of resources in accordance with the law), and autonomy (allowing free, informed, and deliberate decisions). Contested issues, e.g., (in) actionability, are ranked under beneficence as well as non-maleficence to reflect the theoretical debate and their subjective nature. Societal considerations are interrelated, e.g., sharing test results can lead to support but also stigma and discrimination. The visual overview highlights the tension between clinicians’ responsibility to weigh the benefits and risks and prevent unnecessary suffering, versus individuals’ right to self-determination

In substantiating their arguments, the majority of authors invoked the key principles of medical ethics: beneficence, non-maleficence, justice, and autonomy [14, 15, 17, 19–32, 35–39]. We therefore organized all 26 considerations not only by the context they relate to, but also by the key principle that was most applicable, as visualized in Fig. 2.

## Discussion

We found 26 considerations relevant to disclosing a diagnosis of AD to individuals without dementia. These concerns, constraints, and implications relate to clinical, personal, and societal contexts. Many constitute direct opposites, such as certainty versus uncertainty, reflecting the heated debate among stakeholders. This duality is concordant with findings from a recent study on patients’ views regarding early AD diagnosis, reporting not only great variety between individuals but also profound ambivalence within individuals [41]. For example, while a diagnosis can provide certainty on what is going on, it can also bring uncertainty on what to expect. Thus, it may not have to be either one way or the other, as both sides can be true to some extent, and perspectives may change over time. This illustrates the ardent need of empirical evidence and clinical recommendations on a biomarker diagnosis of AD. Since market access has been granted to a first disease-modifying therapy [10], the urgency is even greater, as practitioners, patients, and society are presented with novel opportunities and challenges.

Particularly with respect to clinical validity, the comparison of statements was hampered by a disparity of definitions. In the absence of a gold standard, authors evaluated the accuracy of the biomarker framework [4] according to various views on the true state of AD. Findings were based on different criteria of clinical symptoms, pathological findings, and/or biological changes. While all models have value, they are not interchangeable. Moreover, studies suggest the scientific dissensus on nosology and the shifting meaning of AD create confusion [42–45]. This emphasizes the need for a common concept and language of AD [46].

An underlying and fundamentally contested conundrum is whether individuals with normal cognition, but abnormal biomarkers are ill. Based on research criteria, they have AD but judging by clinical standards they are not sick [21, 27, 30–32, 38, 47]. Two of the included papers evaluate the conceptual validity according to theories of health and disease [26, 34]. The authors reasoned that the signature of amyloid and tau does not represent a singular disease, nor a statistical deviation from normal aging in older people. They concluded that people without symptoms should not be diagnosed as “patients-in-waiting,” but considered persons at-risk [34]. Yet this is not about screening unsuspecting populations. Neither is the phase before dementia entirely without symptoms; individuals present at memory clinics because they experience symptoms in their daily lives, albeit subtle or mild [48, 49]. They wish to learn what is wrong, and they have a right to know. In the field of oncology, it is common to diagnose patients with cancer (in situ), regardless of signs or complaints. The same goes for conditions like hypertension and diabetes mellitus [50, 51]. An apparent difference is the lack of disease-modifying interventions for AD. Some ethicists apply a “pragmatic view” to AD, stating that without preventive medication early detecting may do more harm than good [34]. This view might change with the recent conditional approval of a first disease-modifying treatment by the FDA.

Overall, the identified literature tended to concentrate on putative adverse implications. Notably, repeatedly mentioned worries about conforming to stereotypes or nocebo reactions were substantiated by evidence from a single study on disclosure of genetic risk [52]. Although inherited susceptibility is beyond the scope of our review, extensive research on the impact of revealing an increased probability [53–60] or absolute certainty [61–63] of developing dementia has demonstrated that catastrophic outcomes are rare, knowledge of the test results does not affect cognition, and participants also perceive benefits. So far, evidence on disclosing biomarker information is limited, but the few available studies suggest it is safe and actionable [64–69]. However, stigmatization and discrimination are concerns that need further scrutiny [70–72]. More importantly, it should be noted these findings are based on a selection of individuals, willing to participate and learn their disposition to develop dementia. There is a lack of racial, ethnical, cultural, social, economic, and environmental diversity in study populations [73]﻿. More empirical research is required, evaluating both harms and benefits, taking perspectives of individuals from all groups into account.

The key principles of medical ethics, i.e., beneficence, non-maleficence, justice, and autonomy, were frequently invoked to decide whether a predementia diagnosis of AD is justified. However, applying the “four principle approach” may unduly simplify a complicated matter [74]. The framework relies on the notion of a common morality, while the interest, motivation, and implications of biomarker testing are inherently deeply personal [75]. Yet patients’ perspectives and circumstances are under-represented in the theoretical discourse. Rather than risking paternalism by imposing the moral right to know or not know on all, we need a tailored approach in clinical settings to respect the values of each individual. Future research should illuminate which personal factors influence people’s preferences for medical information, as well as the psychological and social implications of disclosing test results. It is pivotal to engage and educate all stakeholders to enable informed (and shared) decision-making and empower individuals in choosing what is best for them. This becomes especially relevant in light of the development of low-cost blood tests [76, 77], advances in risk-reducing lifestyle programs [3, 78–81], and progress on disease-modifying therapies [82–85].

### Strengths and limitations

Our systematic review provides an in-depth overview of considerations regarding a diagnosis of AD before dementia. Strengths are our broad query to include publications from various disciplines (including medical, ethical, psychological, social, and legal), strict adherence to PRISMA guidelines, and use of state-of-the art methodology to inductively analyze the literature. Among the potential limitations is the restriction to articles in English presenting theoretical data. A next step is an inventory of empirical evidence in the clinical, personal, and societal contexts to compare expectations to experiences and identify gaps in knowledge. Immediate requirements include devising educational materials for the general public, protocols for clinical practice, supportive services for patients, and legislation to protect their rights [86]. The identified considerations offer helpful starting points to prepare for a future with precision medicine and prevention of AD.

## Conclusions

Diagnosing AD in individuals without dementia involves diverse and often opposing considerations, related to a clinical, personal, or societal context. The theoretical literature tended to focus on adverse impact and rely on the notion of a common morality while the motivation for, and implications of, biomarker testing are deeply personal. Our findings provide a starting point for memory clinic specialists, such as neurologists, geriatricians, and psychiatrists, to discuss a biomarker-based diagnosis with their patients, to enable shared and informed decision-making, which will become even more relevant in light of the conditional approval of a first disease-modifying drug for AD.

## Data Availability

The datasets used and/or analyzed during the current study are available from the corresponding author on reasonable request.
